# Effect of family intervention on relapse rate of Chinese patients with alcohol-dependent

**DOI:** 10.3389/fpubh.2024.1327844

**Published:** 2024-05-22

**Authors:** Yi-Jia Wang, Shu-Si Tang, Guang-Dong Chen, Jin-Hong Xia, Li-Na Wang, Huan-Le Zhang

**Affiliations:** Wenzhou Seventh People’s Hospital, Wenzhou, China

**Keywords:** alcohol dependence, intimacy and adaptability, family intervention, relapse rate, family intimacy and adaptability

## Abstract

**Objective:**

This study explored the impact of a family intervention on the relapse rate of Chinese patients with alcohol dependence.

**Methods:**

A total of 151 male patients with alcohol dependence who were discharged from the Substance Dependence Department of the Wenzhou Seventh People’s Hospital from January to December 2020 were selected. They were divided into the control (*n* = 73) and experimental (*n* = 78) groups. Patients in both groups received routine alcohol cessation treatment. Moreover, patients in the experimental group were followed up by a professional psychiatrist to carry out individual family intervention. The Family Function Rating Scale (FAD), a Self-made general information questionnaire, and the Chinese version of the Family Intimacy and Adaptability Scale (FACESI-CV) were performed. Re-drinking rate and readmission rate were assessed.

**Results:**

Family intervention could reduce relapse rate (31, 39.74%) and rehospitalization (27, 34.62%) compared with the control group. After family training, FAD factor scores were improved in the experiment group in comparison with the control group. Family training improved communication (18.2 ± 3.7), role (20.8 ± 2.5), emotional response (10.8 ± 1.8), emotional involvement (13.7 ± 1.2), behavioral control (19.8 ± 1.2), and overall functionality (23.5 ± 2.1) in the experiment group in comparison with the control group. After family training, intimacy (70.5 ± 8.7) and adaptability (64.1 ± 6.9) in the experiment group was higher than in the control group. After family intervention, Michigan Alcohol Dependence Scale (MAST) (9.21 ± 0.68) and Short-Form 36 (SF-36) (80.32 ± 4.47) in the experiment group were higher than the control group.

**Conclusion:**

Family intervention for families of patients with alcohol dependence can improve their family function, increase their family intimacy and adaptability, and reduce the rate of relapse.

## Introduction

Alcohol dependence is a chronic mental disorder characterized by a high rate of relapse (1). Research shows that the relapse rate of patients with alcohol dependence after hospitalization is as high as 58 to 66% within 3 months after discharge (2). Even with intensified psychological intervention, there is still a high relapse rate after discharge (3). Therefore, clarifying the relevant factors of relapse is of great clinical significance in reducing the risk of alcohol dependence relapse (4). Previous studies have mostly explored the issue of alcohol dependence relapse from the perspective of psychological and social factors, with few studies on biological predictive indicators and family factors related to alcohol dependence relapse (5–7).

Alcohol dependence syndrome is a serious social and medical problem today (8). According to the 2010 global disease burden ranking published by Lancet magazine, alcohol has rapidly climbed from 6th place to 3rd place after hypertension and smoking among all disease risk factors, making the situation particularly worrying (9). Alcohol dependence syndrome caused by drinking is also an important psychosocial problem, which is one of the common diseases in psychiatry (10). It has a high incidence rate, great harm, easy recurrence, and other characteristics, causing a serious economic and psychological burden on families and society (11). Research has shown that patients with alcohol dependence syndrome generally have depressive emotions and are not easily identifiable, increasing the risk of suicide for patients (12).

The problem of alcohol dependence is a problem for the entire family, and support from the family and society is an important part of treatment (13). Therefore, health education should be provided to the family members of patients, including knowledge related to alcohol dependence, symptoms and prevention of relapse, psychological guidance, etc. based on the knowledge level and family status of family members (14). This will help them change their attitude towards alcohol-dependent individuals, not pose discrimination against patients, learn to communicate and communicate with patients, maintain family harmony, and increase the stability of their condition (15). Research has shown that family support can improve the quality of life of patients with alcohol-induced mental disorders, reduce the occurrence of anxiety and depression, reduce the frequency of alcohol consumption, and reduce the occurrence and severity of mental symptoms (16, 17). It can also improve patients’ ability to respond to adverse events, timely alleviate psychological crises, and fundamentally achieve the goal of eliminating bad drinking habits (18). However, there was no report on the effect of family intervention on the relapse rate of alcohol dependence in Chinese patients. Therefore, we aimed to explore the impact of a family intervention on the relapse rate of Chinese patients with alcohol dependence.

## Participants and methods

### Ethics

This study was approved by the Ethics Committee of the Wenzhou Seventh People’s Hospital.

### Participants

All patients have signed an informed consent form. A total of 151 male patients with alcohol dependence who were discharged from the Substance Dependence Department of the Wenzhou Seventh People’s Hospital from January to December 2020 were selected. Inclusion criteria: (1) Meets the diagnostic criteria for mental and behavioral disorders caused by alcohol use in ICD-10; (2) Age ≥ 18 years old; (3) Able to understand the questionnaire content and willing to cooperate in completing it. Exclusion criteria: (1) accompanied by severe organic diseases of the body; (2) Organic mental disorders cannot independently complete the questionnaire content evaluation; (3) Patients with other types of mental disorders such as anxiety, depression, and schizophrenia before alcohol dependence. They were randomly divided into an experimental group of 78 cases and a control group of 76 cases using a simple sampling method.

### Intervention methods

Patients in both groups received routine alcohol cessation treatment. Patients in the control group did not receive family intervention and only received general guidance from the supervising physician upon discharge. Patients in the experimental group were followed up one year by a professional psychiatrist to carry out individual family intervention on the patients and their families. The propaganda lectures were given once a month for 45 min, a total of 12 times. Individual family intervention mainly included the family structure and function, close relationship between family members, and communication with each other. The propaganda lecture mainly discussed the harm of alcohol consumption to the heart, liver, stomach, and nervous system, as well as its impact on mental activities through online. The economic burden caused by alcohol consumption and its impact on work, family, and interpersonal relationships were also discussed. Professional knowledge training on alcohol dependence and its prevention and relapse were provided to patients’ families, especially those living with patients (at least one family member), to familiarize them with the prevention, occurrence, and clinical manifestations of alcohol dependence and knowledge of treatment. Moreover, online discussion with the doctors was performed to mobilize the patient’s other relatives and friends to care for, support and supervise him, and to control drinking opportunities, develop a plan to quit alcohol and develop rewards and punishments.

### Assessment tools

The Family Function Rating Scale (FAD) consists of items that summarize family function into the following seven aspects (19): problem-solving, communication, role, emotional response, emotional intervention, behavioral control, and overall function. The score range for each item is 1–4 points. The author of the scale has repeatedly tested the reliability and validity of FAD, indicating that FAD has good reliability and validity. The higher the score, the worse the family function.

Self-made general information questionnaire: including gender, age, education level, average daily alcohol consumption, drinking years, and disease course. Michigan Alcohol Dependence Scale (MAST) (20): this scale includes 5 dimensions, including alcohol consumption basis, the impact of alcohol consumption on work, family, and health, with a total of 24 items. Each entry contains 2 options of ‘yes’ or’ no ‘, each with a score of 1 or 0. The total score range is 0–24 points, and the higher the score, the more severe the alcohol dependence. When the score on the equivalence table is ≥8 points, it indicates a significant alcohol dependence and is recorded as a patient’s relapse. SF-36 Quality of Life Scale (21): This scale was developed by the American Medical Research Group, and the Chinese version was translated and introduced by Li Lu and others. It is widely recognized and widely used internationally. This scale has 8 dimensions, including physiological function, physiological function, physical pain, overall health, vitality, social function, emotional function, and mental health. There are a total of 36 items, and the standard score is obtained by converting the original score, with a score range of 0–100 points. The higher the score, the better the quality of life.

The Chinese version of the Family Intimacy and Adaptability Scale (FACESI-CV) is a self-evaluation scale that includes 2 subscales, Intimacy, and Adaptability, with a total of 30 items (22). The answer score for each item is 1–5 points, indicating the emotional connection between family members; Adaptability refers to the ability of the family system to change accordingly to the family situation and problems that arise at different stages of family development. The scale is rated in five levels: “not,” “occasionally,” “sometimes,” “often,” and “always.” Participants are required to answer their actual feelings about their family status. Both groups of patients were evaluated using the above two scales at enrollment and 1 year after discharge.

Re-drinking rate and readmission rate: two groups of patients were followed up one year after discharge. Those who met the diagnostic criteria for alcohol dependence syndrome or scored ≥6 points on the Michigan Alcohol Dependence Survey (summation method) test questions were judged as re-drinking or readmission for treatment.

### Data collection

Before discharge and 1 year after discharge, patients’ alcohol dependence and quality of life were evaluated when they returned to the follow-up hospital. A uniformly trained management team distributed questionnaires to the two groups of patients, with unified guidance language and on-site filling. After checking without errors, the questionnaires were collected. The location for data collection is set at the outpatient clinic, and data collection will be completed within 1 week after the intervention.

### Statistical analysis

All data were analyzed by SPSS 26.0. Counting data were expressed as numbers and percentages, and compared by using the *χ*^2^ test for inter-group comparison, measurement data were expressed as mean ± standard deviation (SD), and a *t*-test was used for inter-group comparison. *p* < 0.05 was statistically significant.

## Results

### General condition and laboratory test results

There were no statistically significant differences in age (95%CI = −1.989–4.380), BMI (95%CI = −1.116–1.370), years of education, marital status, smoking history, and drinking family history between the control and experimental groups (*p* > 0.05, Table 1).

**Table 1:** General condition.

Groups		Control	Experiment	95%CI
Number		73	78	
Years old		42.85 ± 10.12	41.65 ± 9.66	−1.989 ~ 4.380
BMI(kg/m^2^)		23.61 ± 3.74	23.48 ± 3.98	−1.116 ~ 1.370
Years of Education	<6 years	8	9	
	6–12 years	11	13	
	>12 years	54	56	
Marital status	Unmarried	5	6	
	Married	56	59	
	Divorce, remarriage, or bereavement	12	13	
Smoking history	Smoke	63	66	
	Stopped smoking	4	5	
	No Smoking	6	7	
Drinking family history	Positive	14	16	
	Negative	59	62	

### Drinking status and laboratory test results

There were no statistically significant differences in starting drinking age, addiction time, daily alcohol consumption, Hcy, Folic acid, vitamin B12, triglyceride, cholesterol, blood glucose, total bilirubin, MCV, and Hb levels between the control and experimental groups (95% CI were −2.164–0.587, −0.664–0.308, −1.592–1.182, −2.937–4.750, −0.472–0.270, −42.757–9.967, −0.789–0.277, 0.161–0.848, −0.315–0.704, −3.994–3.015, −0.853–2.074, −4.529–6.124, *p* > 0.05, Table 2).

**Table 2:** Drinking status and laboratory test results.

Groups	Control	Experiment	95%CI
Starting drinking age (years)	22.3 ± 4.8	23.1 ± 3.6	−2.164 ~ 0.587
Addiction time (years)	5.4 ± 1.2	5.6 ± 1.7	−0.664 ~ 0.308
Daily alcohol consumption (Standard Cup)	15.6 ± 4.5	15.8 ± 4.1	−1.592 ~ 1.182
Hcy (μmol/L)	27.8 ± 12.4	26.9 ± 11.5	−2.937 ~ 4.750
Folic acid (ng/mL)	3.4 ± 1.2	3.5 ± 1.1	−0.472 ~ 0.270
Vitamin B 12 (pg/mL)	402.5 ± 86.7	418.9 ± 77.2	−42.757 ~ 9.967
Triglyceride (mmol/L)	1.4 ± 0.5	1.3 ± 0.6	−0.789 ~ 0.277
Cholesterol (mmol/L)	5.4 ± 1.2	4.9 ± 0.9	0.161 ~ 0.848
Blood glucose (mmol/L)	5.7 ± 1.8	5.5 ± 1.3	−0.315 ~ 0.704
Total bilirubin (μmol/L)	22.6 ± 10.2	23.1 ± 11.5	−3.994 ~ 3.015
MCV (%)	98.2 ± 4.8	97.6 ± 4.3	−0.853 ~ 2.074
Hb (g/L)	134.6 ± 16.6	133.8 ± 16.5	−4.529 ~ 6.124

### Comparison of relapse status between two groups of patients

Family intervention improved the relapse rate (31, 39.74%) and rehospitalization rate (27, 34.62%) in comparison with the control group (Table 3).

**Table 3:** Comparison of rehydration status between two groups of patients.

Groups	Control	Experiment
Number	73	78
Relapse rate	42 (57.53%)	31 (39.74%)
Rehospitalization	36 (49.32%)	27 (34.62%)

### Comparison of FAD factor scores

After family training, FAD factor scores were improved in the experiment group in comparison with the control group (Table 4). Family intervention ameliorated communication (18.2 ± 3.7), role (20.8 ± 2.5), emotional response (10.8 ± 1.8), emotional involvement (13.7 ± 1.2), behavioral control (19.8 ± 1.2), and overall functionality (23.5 ± 2.1) compared with the control group (Table 4).

**Table 4:** Comparison of FAD factor scores.

FAD	Control		Experiment	
	Before training	At training	Before training	At training
Problem-Solving	11.6 ± 2.6	10.9 ± 2.2	11.4 ± 2.3	10.3 ± 1.9
Communicate	22.9 ± 3.6	21.5 ± 3.5	22.7 ± 2.8	18.2 ± 3.7^*#^
Role	27.9 ± 3.8	27.6 ± 3.2	28.1 ± 3.1	20.8 ± 2.5^*#^
Emotional response	15.2 ± 1.8	14.7 ± 1.9	15.5 ± 2.7	10.8 ± 1.8^*#^
Emotional involvement	18.8 ± 2.2	18.1 ± 2.7	18.6 ± 2.3	13.7 ± 1.2^*#^
Behavioral control	25.4 ± 2.7	23.8 ± 2.1	24.6 ± 2.4	19.8 ± 1.2^*#^
Overall functionality	31.2 ± 3.7	29.4 ± 3.3	31.5 ± 4.2	23.5 ± 2.1^*#^

^*^p < 0.05, compared with the values before training. #*p* < 0.05, compared with the values in the control group at training.

### Comparison of FACES II-CV scores

After family intervention, intimacy (70.5 ± 8.7) and adaptability (64.1 ± 6.9) in the experiment group were ameliorated in comparison with the control group (Table 5).

**Table 5:** Comparison of FACES II-CV scores.

FACES II-CV	Control		Experiment	
	Before training	At training	Before training	At training
Intimacy	57.1 ± 10.5	57.8 ± 6.7	57.6 ± 9.2	70.5 ± 8.7^*#^
Adaptability	48.3 ± 8.9	50.3 ± 5.6	49.1 ± 9.1	64.1 ± 6.9^*#^

^*^p < 0.05, compared with the values before training. #*p* < 0.05, compared with the values in the control group at training.

### MAST score and SF-36 score

After family intervention, MAST (9.21 ± 0.68) and SF-36 (80.32 ± 4.47) in the experiment group were ameliorated than those in the control group (Table 6).

**Table 6:** MAST score and SF-36 score.

Group	Control		Experiment	
	Before training	At training	Before training	At training
MAST	15.63 ± 1.42	11.36 ± 0.84	15.37 ± 1.38	9.21 ± 0.68^*#^
SF-36	55.69 ± 7.22	63.02 ± 5.32	55.91 ± 7.12	80.32 ± 4.47^*#^

^*^p < 0.05, compared with the values before training. #*p* < 0.05, compared with the values in the control group at training.

## Discussion

Alcohol dependence syndrome is a recurrent and chronic brain disease caused by central nervous system poisoning caused by habitual excessive drinking (23). On the one hand, alcohol dependence will damage the patient’s physical and mental health, on the other hand, it will also bring great burden to the patient’s family, and even cause family breakdown in severe cases, which will lead to a series of social problems (24). Re-drinking rate is a key indicator to judge the therapeutic effect of patients with alcohol dependence syndrome (25). In this study, we explored the effect of family intervention on the recurrence rate of patients with alcohol dependence. Our results show that family intervention for families of patients with alcohol dependence can improve their family function, increase family cohesion and adaptability, and reduce the recurrence rate.

The education level, emotional life and marital status of patients with alcohol dependence syndrome are the main factors leading to their re-drinking (26). Under good and harmonious family relations, patients with alcohol dependence syndrome often have a low rate of re-drinking and a high success rate of abstinence (27). It is reported that patients with alcoholism are more often raised by single parents, they are more likely to have alcohol-dependent parents, and their relationship with their parents is more often impaired (28). Familiarity with the patient’s family status is of great significance for the treatment of alcohol dependence syndrome. Involving the family members of alcohol dependent patients will definitely help to provide support for patients and help them continue to receive treatment. Family intervention can also help prevent problems for spouses or children of alcoholics (29). At the beginning of the study, most families were characterized by poor communication patterns, lack of mutual warmth and support, poor role functioning, lack of leadership and abusive spouses (30). The spouses of alcoholics expressed greater dissatisfaction with all areas of family functioning. After family intervention, these families showed greater satisfaction with family functions, such as free and open communication, mutual warmth and support, becoming ideal role models, showing good leadership, cohesion and responsibility sharing. A randomized trial in the United States showed that brief family therapy interventions promoted continuous care for hospitalized patients with alcohol dependence (31).

This study showed that family intervention can effectively improve the recurrence rate and readmission rate of patients with alcohol dependence. In addition, family intervention can also significantly improve family function, internal communication, emotional response, emotional mediation, behavior control and overall function. Studies have also shown that community reinforcement and family training are effective in treating significant others in the treatment of alcohol-dependent individuals in terms of participation, improving mental health and family cohesion (32). A 6 months follow-up study of Indian alcohol dependents showed that when both participants and a family member were included in the intervention, the reduction of alcohol consumption, drinking days and family, occupational and economic dysfunction days was significantly better than that of only participants included in the intervention (33). This shows that after the intervention, family members can better express their emotions, enhance mutual trust among family members, reduce the occurrence of stress events within the family, and improve the family support system.

After 1 year of follow-up, it was found that the intervention score was significantly higher than the control group, and the difference was statistically significant, indicating that the research group had better family intimacy and adaptability than the control group. Family intervention for patients with alcohol dependence and their families can improve the family environment, and increase family intimacy and adaptability, which is consistent with relevant research results. The alcohol relapse rate and readmission rate in the intervention group were significantly lower than those in the control group, indicating that family intervention can reduce the relapse rate of alcohol-dependent individuals.

This study also has some limitations. First, the sample size is small. In addition, this study is a single-center study. Moreover, the follow-up time is relatively short. Furthermore, only males were included in this study. In the future, studies with larger sample size, randomized and longer follow-up time will provide more information on the efficacy of family intervention in the management of patients with alcohol dependence syndrome.

## Conclusion

Through family intervention for patients with alcohol dependence and their families, their family intimacy and adaptability are increased, their family function is improved, and the entire family system operates more effectively, providing more support and supervision to patients with alcohol dependence, and reducing the relapse rate.

## Data availability statement

The original contributions presented in the study are included in the article/supplementary material, further inquiries can be directed to the corresponding author.

## Author contributions

Y-JW: Data curation, Formal analysis, Investigation, Validation, Visualization, Writing – original draft, Writing – review & editing. S-ST: Formal analysis, Methodology, Resources, Visualization, Writing – review & editing. G-DC: Conceptualization, Data curation, Methodology, Resources, Writing – review & editing. J-HX: Conceptualization, Data curation, Formal analysis, Methodology, Writing – review & editing. L-NW: Data curation, Investigation, Supervision, Visualization, Writing – review & editing. H-LZ: Formal analysis, Investigation, Software, Supervision, Writing – review & editing.
